# On the Manna of Sinai, and the Manna of Syria

**Published:** 1862-08

**Authors:** M. Berthelot


On the Manna of Sinai, and the Manna of Syria.—*By M. Berthelot.*—"They took their journey from Elim, and all the congregation of the children of Israel came unto the wilderness of Sin, which is between Elim and Sinai. . . . . And the whole congregation of the children of Israel murmured against Moses and Aaron; and the children of Israel said unto them . . . . Why have ye brought us forth into this wilderness, to kill this whole assembly with hunger? Then said the Lord unto Moses: Behold I will rain bread from heaven for you. . . . . And behold upon the face of the wilderness there lay a small round thing, as small as the hoar frost on the ground. And when the children of Israel saw it, they said one to another: *Manna?* which signifies: What is it? . . . . And the house of Israel called the name thereof Manna. . . . . The taste of it was like honey. . . . And the children of Israel did eat Manna forty years. . . . . They did eat Manna, until they came to the borders of the land of Canaan."—*Liber Exodi*, cap. xvi.

What is the substance described in the preceding recital, which figures so much in the history of the Hebrew nation, and the name of which serves as a type of a multitude of natural sugary substances? Can it be likened to any saccharine matter known in the present day? This is a very controverted question. Two principal opinions are current on this point: the one regards manna as a sugary exudation supplied by various shrubs, principally by the *Alhagi Maurorum* (Tourn.), a kind of saintfoin; the other opinion likens the manna of the Hebrews to a species of cryptogamous plant. In these days the origin of the manna gathered at Sinai may be regarded as settled, according to the researches made upon the spot by MM. Ebrenberg and Hemprich. "Manna," says

Ebrenberg, "is found at the present time in the mountains of Sinai; it falls upon the earth from the regions of the air (that is to say, from the top of a shrub, and not from the heavens.) The Arabs call it *Man.* The indigenous Arabs and the Greek monks collect it and eat it with bread in the same way as honey. I have seen it fall from the tree, I have gathered it, brought it myself to Berlin with the plant and the remains of the insect." This manna flows from the *Tamarix mannifera* (Ehr.) Like a number of other mannas, it is produced under the influence of the puncture of an insect, the *Coccus Manniparus* (H. and Ehr.)

If the origin of the Sinai manna be thus established, it is not so with its chemical nature. And this is a subject the more interesting, because the chemical analysis can alone explain its character as a nutritive agent. The result of my researches among sugary substances has led me to make some experiments in this respect. I have operated upon the following substance, the one identical with, the other analogous to, the manna of Sinai:—1st. The Manna of Sina; 2d. The Manna of Syria, or rather of Kurdistan.

1st. The Manna of Sinai.

The specimen was given to me by M. Decaisne; it was produced from the *Tamarix mannifera;* it had been gathered and brought home by M. Leclerc, who accompanied the Princess of Orleans in a voyage to the East (1859—1860.)

This manna presents the appearance of a yellowish syrup, thick, and containing vegetable remains. It contains cane sugar, interverted sugar, dextrine, and water. The weight of the water was about a fifth of that of the whole mass. Its composition, after the removal of the water and vegetable remains, was as follows:

| | |
|---|---|
| Cane sugar | 55 |
| Interverted sugar (levulose and glucose) | 25 |
| Dextrine and analogous substances | 20 |
| | 100 |

2d. Manna of Kurdistan.

The specimen was given to me by M. L. Soubeiran; it had been sent to Paris by Dr. Gaillardot. It had been gathered on the mountains of Kurdistan, to the N. E. of Mossoul. Subjoined is the information on this subject in a letter addressed to M. Gaillardot by M. Barré de Lancy, then Chancellor to the Consulate of France at Mossoul. This manna "falls without distinction on every plant in July and August, but not every year; there has been very little for the last three years. It is gathered by cutting the branches, which are left to dry for two or three days in the sun, when they are shaken and the manna is obtained, which falls like dust. The Kurds use it without purifying it. They mix it with dough, and even with meat." The matter presents itself under the form of a pasty mass, almost solid, impregnated with vegetable matter, and especially with the leaves of the nut-gall tree. It contains cane sugar, interverted sugar, dextrine, water, and, lastly, a small quantity of green waxy matter. This is the composition of the part soluble in water:

| | |
|---|---|
| Cane sugar | 61. |
| Interverted sugar (levulose and glucose) | 16.5 |
| Dextrine and analogous substances | 22.5 |
| | 100.0 |

From the preceding results, we see that the manna of Sinai and that of Kurdistan are essentially composed of cane sugar, dextrine, and the products of the alteration, without doubt, of these two principles. Their composition is almost identical, a result the more belonging to two extremely different species. Nevertheless, this phenomenon is not without analogy. Thus we know that the singular, because the plants which produce these two mannas honey gathered by bees from very different flowers possesses a composition almost identical. This is not the only comparison which may be made between honey and the manna in question. Not only do insects concur equally in the formation of honey and that of the manna of Sinai, but further, this manna, as well as honey, if composed of cane sugar and interverted sugar; the manna of Sinai contains, beyond this, dextrine, and the products of its alteration.

If we return now to the historical recital of the manna of Sinai, it is easy to explain the use of this substance as a food. In short, it is a true honey, perfected by the presence of dextrine. We see, at the same time, that the manna of Sinai does not suffice as an aliment, since it does not contain the nitrogenous principle. Therefore, animal food is associated with it, as well in the actual usage of the Kurds as in the biblical narratives.—See Exodus xvi. 8-13.—*Lond. Pharm. Journ., Nov.,* 1861, *from Comptes Rendus.*

